# Exploring Photocatalytic, Antimicrobial and Antioxidant Efficacy of Green-Synthesized Zinc Oxide Nanoparticles

**DOI:** 10.3390/nano15110858

**Published:** 2025-06-03

**Authors:** Sabina Shrestha, Laxmi Tiwari, Sujan Dhungana, Jasana Maharjan, Devendra Khadka, Allison A. Kim, Megh Raj Pokhrel, Janaki Baral, Mira Park, Bhoj Raj Poudel

**Affiliations:** 1 Department of Chemistry, Tri Chandra Multiple Campus, Tribhuvan University, Kathmandu 44600, Nepal; sabina.shrestha@trc.tu.edu.np (S.S.); tiwarilaxmi090@gmail.com (L.T.); sujandhungana73@gmail.com (S.D.); janaki.baral@trc.tu.edu.np (J.B.); 2 Department of Chemistry, Padma Kanya Multiple Campus, Tribhuvan University, Kathmandu 44600, Nepal; jasana.maharjan@pkmc.tu.edu.np; 3 Department of Chemistry, Tribhuvan Multiple Campus, Tribhuvan University, Tansen 32500, Nepal; devendra.khadka@tmc.tu.edu.np; 4 Central Department of Chemistry, Tribhuvan University, Kirtipur, Kathmandu 44600, Nepal; megh.pokhrel@cdc.tu.edu.np; 5 Department of Healthcare Management, Woosong University, Daejeon 34606, Republic of Korea; allisonkim@wsu.ac.kr; 6 Carbon Composite Energy Nanomaterials Research Center, Woosuk University, Wanju-Gun, Chonbuk 55338, Republic of Korea; 7 International Joint Research Institute for Future Energy, Woosuk University, Wanju-Gun, Chonbuk 55338, Republic of Korea; 8 Department of Automotive Engineering, Woosuk University, Wanju-Gun, Chonbuk 55338, Republic of Korea; 9 Woosuk Institute of Smart Convergence Life Care (WSCLC), Woosuk University, Wanju-Gun, Chonbuk 55338, Republic of Korea

**Keywords:** *Aloe vera*, antibacterial properties, antioxidant activity, photocatalysis, environmental remediation, nanotechnology

## Abstract

*Aloe vera* is effectively utilized to synthesize zinc oxide nanoparticles (Av-ZnO NPs), providing an alternative to traditional chemical and physical methods. This sustainable approach minimizes the environmental impacts and enhances their compatibility with herbal ecosystems. We comprehensively analyzed the optical, structural, morphological, and catalytic properties of Av-ZnO NPs using various analytical methods. The results indicated that the nanoparticles primarily exhibited a spherical shape. X-ray diffraction (XRD) revealed the successful formation of a highly crystalline hexagonal wurtzite structure, with an average size estimated at 12.2 nm. The antimicrobial properties of the Av-ZnO NPs indicated moderate antibacterial effectiveness. Using the DPPH free radical scavenging method, we evaluated the antioxidant properties, where the Av-ZnO NPs exhibited improved the radical scavenging efficiency, reflected by a lower IC_50_ value compared to the plant extract. Additionally, we assessed the photocatalytic functionality through the degradation of methylene blue (MB) dye, finding that the Av-ZnO NPs achieved approximately 82.43% degradation in 210 min, demonstrating their potential for environmental remediation. These findings suggest that green-synthesized ZnO NPs could play a noteworthy role in various nanotechnology applications and biomedical fields, while also promoting environmental sustainability.

## 1. Introduction

Tiny particles with lengths extending from 1 to 100 nanometers are known as NPs. If at least one dimension of a material is within this nanoscale size range, it is referred to as a nanomaterial [1]. Metal NPs are imperative in nanotechnology and nanoscience because of their numerous conceivable applications [2]. Nanotechnology has greatly impacted various scientific fields, such as medicine, materials science, electronics, catalysis, and pollution control, by utilizing the unique physicochemical characteristics of nanomaterials [3,4,5]. The remarkable surface area, when compared to their volumes, as well as the increased chemical reactivity and chemical stability of metal oxide NPs have increased their prominence and applications in different fields [6,7,8,9]. Because of the wide 3.37 eV band gap and binding energy of approximately 60 meV, ZnO stands out among the materials listed [10], with versatile applications in antibacterial treatment, ultraviolet protection, photocatalysis, drug delivery, and promoting environmental sustainability [11,12,13,14].

Researchers employ different techniques to synthesize NPs. Conventional synthetic routes for the production of ZnO NPs, such as hydrothermal [15], solvothermal [16], chemical precipitation [17], and microwave-assisted techniques [18], frequently use hazardous chemicals and demand high energy consumption. In natural synthesis techniques, microorganisms such as microbes, organisms, yeasts, algae, and plant extracts have been utilized [19]. Contrary to established chemical and physical synthesis protocols, the eco-friendly technique of producing metal oxide NPs is gaining popularity and recognition. This strategy is economical and energy-efficient and facilitates the large-scale generation of NPs by utilizing plant extracts, making the process relatively simple and sustainable [20]. The *Aloe vera* plant, recognized for its medicinal properties, has recently drawn interest as an eco-friendly bio-template for synthesis. *Aloe vera* extract is abundant in phytochemicals, including polysaccharides, phenolic compounds, and amino acids [21]. Due to their carboxyl, hydroxyl, and amino functional groups, these substances play a crucial role in stabilizing and reducing metal ions.

Green-synthesized ZnO NPs show a significant antimicrobial effect, primarily the formation of reactive oxygen species (ROS), which compromise microbial cell membranes and lead to their structural disruption [22]. The breakdown of natural toxins like methylene blue is facilitated by their broad band gap and high electron mobility when exposed to visible or UV light. This characteristic makes them essential for water reclamation and gaseous contaminant removal processes [23]. Nevertheless, issues like aggregation and stability can limit their efficacy. To overcome these challenges, ZnO NPs are frequently embedded in biodegradable polymer matrices, such as regenerated cellulose films (RCFs), which enhances their antimicrobial and photocatalytic properties for various applications, including food packaging, medical devices, and environmental cleanup [24,25,26].

Although *Aloe vera* has been utilized in the green route to prepare metal oxide NPs, comprehensive investigations into the multifunctional properties of Av-ZnO NPs remain limited. This research outlines a sustainable synthesis route and systematically evaluates the antimicrobial, antioxidant, and photocatalytic actions. The photocatalytic performance is assessed by degrading MB dye using UV illumination. The integrated assessment of biological and photocatalytic functionalities highlights the possibility of using Av-ZnO NPs for applications in biomedical and environmental fields. While previous studies have explored the green synthesis of ZnO nanoparticles using various plant extracts, we utilize *Aloe vera* extract, which is rich in a unique combination of bioactive compounds. These compounds contribute to both enhanced capping efficiency and improved particle stability. Our study investigates multiple functional properties, such as photocatalytic, antimicrobial, and antioxidant activity, in an integrated manner. The nanoparticles exhibit broad-spectrum antimicrobial activity and significant photocatalytic degradation performance. The biosynthesis method employed is eco-friendly, cost-effective, and scalable.

Biologically derived materials, ranging from plant extracts to proteins, have gained attention as sustainable agents for nanoparticle synthesis and functionalization [27]. While our previous work focused on the *Swertia chirayita*- and *Mentha*-mediated synthesis of ZnO and CuO NPs [28,29], the current study extends this investigation by employing *Aloe vera* extract to compare the impacts of the phytochemical composition on the nanoparticles’ properties and multifunctionality. Although *Aloe vera* has been utilized in the green synthesis of metal oxide nanoparticles, comprehensive investigations into the multifunctional properties of *Aloe vera*-mediated ZnO nanoparticles (Av-ZnO NPs) remain limited. This study presents a sustainable synthesis route for ZnO NPs using *A. vera* leaf extract and systematically evaluates their antimicrobial, antioxidant, and photocatalytic activity. Notably, the photocatalytic performance is assessed through the degradation of methylene blue under UV irradiation. This integrated assessment of their biological and photocatalytic functionalities highlights the potential of Av-ZnO NPs for applications in biomedical and environmental fields.

## 2. Results and Discussion

### 2.1. Phytochemical Screening

The hydro-methanol extract of *Aloe vera* was analyzed for bioactive molecules, with the results presented in Table 1. The test using an alkaline reagent verified the presence of flavonoids [30], whereas the Wagner test indicated a positive result for alkaloids. The froth test indicated the existence of saponins [30]. Glycosides and quinones were not detected, as indicated by negative results in the Salkowski test and the concentrated HCl test, respectively. The ferric chloride test confirmed the presence of tannins and phenols [30]. Furthermore, anthraquinones were present, as evidenced by a positive result in Borntrager’s test.

### 2.2. Biosynthesis Mechanism

**Scheme 1:**
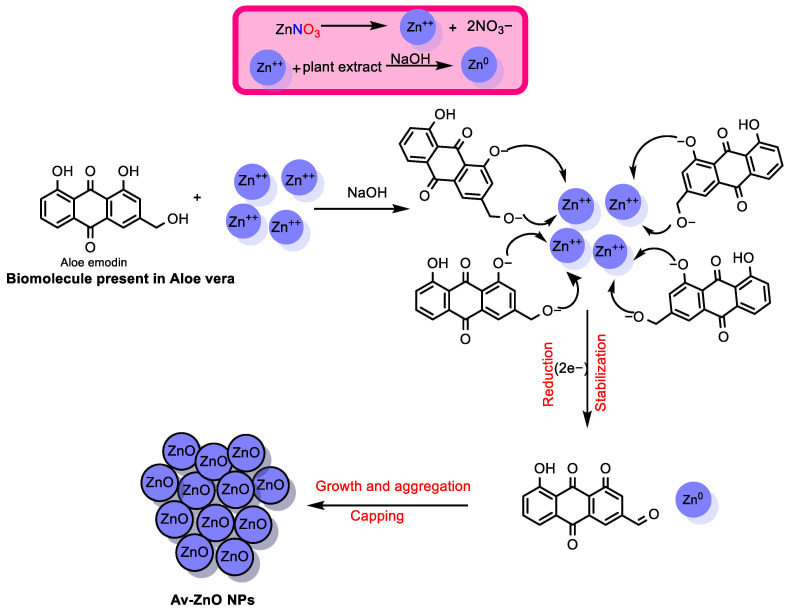
A possible mechanism for ZnO NP formation using *Aloe vera* biomolecules.

The synthesis of ZnO NPs from *Aloe vera* likely follows a three-stage mechanism. Initially, during the activation phase, zinc ions from Zn(NO_3_)_2_ interact with the bioactive compounds in the *Aloe vera* extract. In addition to NaOH, Zn(OH)_2_ is formed more easily. This compound then dehydrates, resulting in the nucleation of ZnO. Smaller NPs combine into larger structures via heterogeneous nucleation and growth during the growth phase, which is driven by Ostwald ripening. The continued reduction of zinc ions also characterizes this stage. Finally, during the termination phase, the NPs achieve their final morphology. Following oxidation, well-dispersed and stable ZnO NPs are obtained. A proposed reaction pathway for this synthesis is illustrated in the accompanying Scheme 1. Similar mechanistic insights have been suggested in various studies [31,32].

This environmentally friendly synthesis method employs redox-active compounds, such as ascorbic acid, polyphenols, and anthraquinones, which function both as reducing agents for Zn^2+^ ions and as stabilizers by binding to the surfaces of the resulting ZnO NPs. Upon biosynthesis, Zn^2+^ is reduced to Zn(0), which rapidly undergoes oxidation to form ZnO nanoparticles. This transformation involves the oxidation of Zn(0) to Zn(II), and, concurrently, the biomolecules (e.g., anthraquinone derivatives) are oxidized to balance the redox process. Unlike earlier approaches, the novelty of this study lies in the use of *Aloe vera* extract as a multifunctional component, serving simultaneously to reduce metal ions and to stabilize the NPs. This dual functionality facilitates better control over the particle size, promotes uniform dispersion, and may enhance the NPs’ biocompatibility. Key advantages of the method include its simplicity and sustainability and the use of renewable plant-based resources.

### 2.3. UV–Visible Spectroscopy

**Figure 1:**
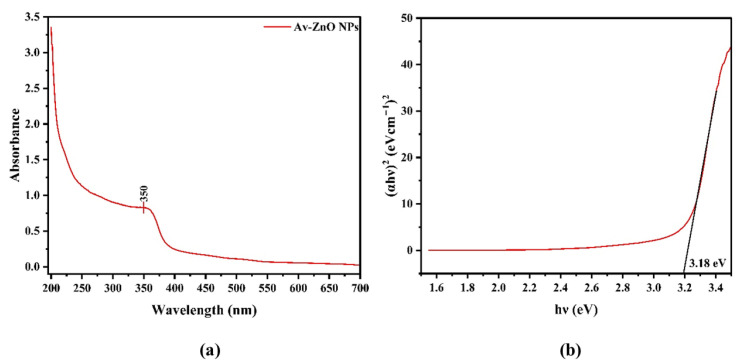
(**a**) Analysis of UV–vis absorption spectrum; (**b**) shows the band gap energy of *Av*-ZnO NPs synthesized from *Aloe vera* extract.

One essential tool in verifying and characterizing the synthesis of Av-ZnO NPs is UV–visible spectroscopy. This method takes advantage of the unique optical properties of ZnO NPs, especially their surface plasmon resonance (SPR) [33]. The SPR effect occurs when the coordinated, rhythmic movement of the surface electrons of NPs is influenced by light. This leads to specific absorption peaks in the UV–visible spectrum, which allow researchers to assess various parameters, including the particle size, shape, and dispersion. The absorption spectrum of the synthesized NPs, presented in Figure 1a, was obtained using a spectrophotometer in the wavelength range of 200–700 nm. The absorption peak at 350 nm verified the formation of ZnO NPs, demonstrating the efficiency of the plant-based method applied in the present study.

The Tauc Equation (1) is employed to derive the band gap energy:

αhν = C (hν − E_g_)^n^ (1)

In Equation (1), the absorption coefficient (α) is related to the photon energy (hv) and a constant (C); the n value is set to 1/2 for semiconductors with a direct band gap. The graph illustrates the band gap of Av-ZnO NPs, determined via the Tauc plot method. The y-axis denotes absorbance, while the x-axis represents energy in electron volts (eV) [34].

A straight line is drawn to intersect the curve at its highest point, which shows a band gap of 3.18 eV (Figure 1b), indicating the NPs’ suitability for visible light absorption.

In addition to determining the optical band gap, it is essential to calculate the positions of the conduction band (E_CB_) and valence band (E_VB_) to evaluate the redox potential of photogenerated carriers. These positions influence the generation of reactive oxygen species, which drive the photocatalytic degradation process, as emphasized in a previous study [35]. The E_CB_ and E_VB_ were calculated using the Butler–Ginley equations (Equations (2) and (3)), as employed in previous studies on semiconductor nanoparticles [36,37]:

(2) $$E_{CB}=X-E_{C}-\frac{1}{2}E_{g}$$

(3) $$E_{VB}=E_{CB}+E_{g}$$

where X is the absolute electronegativity of the semiconductor; E_c_ is the energy of free electrons, commonly taken as 4.5 eV; and E_g_ is the optical band gap energy [38]. For ZnO, the Mulliken electronegativity (X) is reported as 5.79 eV. Using a band gap (E_g_) of 3.18 eV, the conduction band edge is calculated as

E_CB_ = 5.79 − 4.5 − 3.18/2 = −0.30 eV

Accordingly, the valence band edge is

E_VB_ = −0.30 + 3.18 = +2.88 eV

These values indicate that the photogenerated electrons are thermodynamically able to reduce molecular oxygen to superoxide radicals (^•^O_2_^−^), while the holes can oxidize water or hydroxide ions to hydroxyl radicals (^•^OH), both of which are essential for dye degradation.

### 2.4. FTIR Analysis

**Figure 2:**
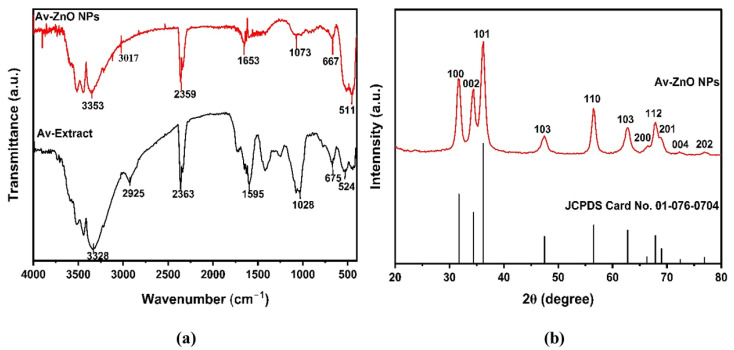
(**a**) FTIR signal examination of Av-ZnO NPs (red) and *Aloe vera* extract (black); (**b**) XRD pattern.

Figure 2a illustrates the FTIR spectrum of the *Aloe vera* extract and *Av*-ZnO NPs. The plant extract displays a broad peak of 3328 cm^−1^, which is related to O–H stretching vibrations from hydroxyl groups found in polyphenols and flavonoids. These groups are known for their strong reducing and capping abilities, which are critical during the biosynthesis of metal oxide NPs. Additionally, C–H stretching vibrations at 2925 cm^−1^ are present in aliphatic compounds, further confirming the presence of organic constituents such as saponins and terpenoids. The peak at 1595 cm^−1^ is associated with C=C stretching vibrations in aromatic rings, consistent with the presence of phenolic compounds. Additionally, C–O stretching and C–H bending vibrations are visible at 1028 cm^−1^ and 675 cm^−1^, respectively, suggesting the presence of alcohols, esters, or ethers [39,40].

The ZnO NPs’ spectrum shows a shift in the O–H stretching band to 3353 cm^−1^, indicating an interaction between the *Aloe vera* extract and the ZnO surface. The C=O functional group manifests as a prominent IR band at 1653 cm^−1^, which indicates that plant phytochemicals have a role in stabilizing ZnO NPs. The broad peak at 511 cm^−1^ corresponds to the stretching of Zn–O bonds. The variations in certain peaks between the ZnO NPs’ spectrum and that of the original plant extract imply that functional groups from the plant extract are vital for the reduction and stabilization of the ZnO NPs. Moreover, the interactions between hydroxyls with carbonyl functionalities in the ZnO particles are crucial in enhancing their stability and minimizing agglomeration [41,42].

The observed spectral differences between the pure *Aloe vera* extract and the synthesized Av-ZnO NPs indicate that the phytochemicals in *Aloe vera* —especially polyphenols, flavonoids, and proteins—are actively involved in the reduction of Zn^2+^ ions to ZnO. These biomolecules also contribute to the capping and stabilization of the NPs. Functional groups such as hydroxyls and carbonyls likely interact with the surface of ZnO, forming a protective organic layer that prevents NP agglomeration. This organic coating not only improves the colloidal stability and regulates the particle size but also results in a biocompatible surface [43,44].

### 2.5. XRD Study

The crystal and phase composition of the ZnO NPs was assessed using XRD. Plant extract-derived NPs exhibited a diffraction pattern from a minimum of 20° to a maximum of 80°. Peaks in the diffraction data emerged at 31.71°, 34.27°, 36.18°, 47.41°, 56.51°, 62.77°, 66.30°, 67.86°, 69.00°, 72.46°, and 76.75°, as shown in Figure 2b. These XRD data suggested Miller indices of (100), (002), (101), (102), (110), (103), (200), (112), (201), (004), and (202), respectively. The existence of these peaks is in line with the characteristics of a hexagonal crystal typical of the wurtzite form, corroborated by JCPDS no. 01-076-0704, which supports the presence of high crystallinity and phase purity in the synthesized NPs [45]. Scherrer’s calculations determined that the average crystal size was 12.2 nm. The absence of supplementary peaks indicated that the sample was exceptionally pure.

The microstrain (ε), dislocation density (δ), and stacking fault are calculated using Equations (4), (5) and (6), respectively [46,47]:

(4) $$\varepsilon =\frac{\beta \;cos\theta }{\begin{array}{c} 4\\ \; \end{array}}$$

(5) $$\delta =\frac{1}{\begin{array}{c} D^{2}\\ \; \end{array}}$$

(6) $$SF=\left(\frac{{2\pi }^{2}}{{45(3tan\theta )}^{\frac{1}{2}}}\right)\beta$$

The equation determines the lattice parameters (a = b, c) for the phase (rhombohedral). The interplanar spacing d for the Miller indices (hkl) is given by Equation (7):

(7) $$\frac{1}{d_{hkl}^{2}}=\frac{4}{3}.\frac{h^{2}+hk+k^{2}}{a^{2}}+\frac{l^{2}}{c^{2}}$$

From XRD, a = b = 3.253 A°c = 5.2130 A°, α = β = 90°, and γ = 120°, with space group = P63mc.

$$Particle\;size\;crystallinity\;Index\left(CI\%\right)=74.5\%$$

Table 2 presents the detailed crystallographic parameters of the synthesized materials, including the crystallite size, dislocation density, strain, structure factor (SF), and relative intensity, derived from individual diffraction peaks. By indicating specific Miller indices and the corresponding FWHM, the table provides accurate crystallite size and strain values for each crystallographic plane, aiding in the identification of anisotropy in crystal growth and lattice distortion. Moreover, the inclusion of the dislocation density and structure factor for each reflection provides deeper insights into the crystal defect density and the intensity contribution of each plane. The synthesized ZnO NPs possess a hexagonal crystal structure, characterized by a pronounced preferential orientation along the (101) crystallographic plane, which exhibits a relative intensity of 100%, reflecting a strong texture. The crystallite sizes are within the nanometer scale, ranging from 7.34 to 16.32 nm, with an average size of 12.2 nm, confirming their nanocrystalline nature. The measured interplanar spacing (d-spacing) falls between 1.239 and 2.817 Å, aligning with typical values for the hexagonal ZnO phase. These nanoparticles also demonstrate low dislocation densities (0.00375–0.01856 nm^−2^) and microstrain values in the range of 0.00212 to 0.00473, indicating high crystallinity and minimal lattice distortions. Additionally, the stacking fault probability lies between 0.00996 and 0.02491, suggesting the presence of moderate planar defects, which are commonly observed in nanostructured materials.

The data in Table 2 are crucial as they provide a more precise and comprehensive understanding of the material’s microstructural characteristics compared to those obtained via other synthesis methods, as listed in Table 3. In comparision to other synthesis methods, the present study demonstrates a finer crystallite size with relatively lower strain and dislocation density, indicating better crystalline quality. Therefore, it substantiates the significance of the current synthesis method by highlighting the superior structural integrity and uniformity of the Av-ZnO NPs.

### 2.6. SEM and EDS Analysis

**Figure 3:**
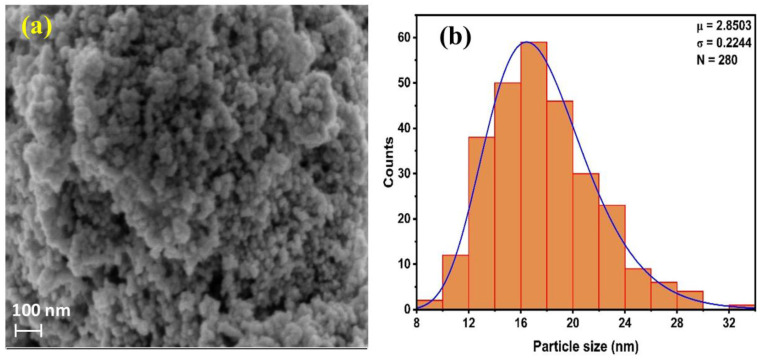
(**a**) SEM image of Av-ZnO NPs; (**b**) histogram showing average particle size distribution.

The SEM image, shown in Figure 3a, reveals slight particle agglomeration, with insufficient separation between them. Additionally, the particles appear to be held together by weak physical forces. The SEM images revealed predominantly spherical and evenly distributed particles. The particle size distribution of the Av-ZnO NPs was determined from the SEM image using the Image J software version 1.54 g. A total of 280 particles were analyzed, and the resulting histogram is presented in Figure 3b. The distribution follows a log-normal trend, with a geometric mean size (μ) of 2.8503 and a standard deviation (σ) of 0.2244, corresponding to an average particle size of approximately 17.78 nm. The histogram reveals a narrow and monodisperse distribution, with most particles ranging between 14 and 20 nm. The slight positive skew indicates uniform nucleation with some degree of particle growth variation, which is typical in green synthesis processes. Such uniformity in size is beneficial for photocatalytic and antimicrobial applications, where a consistent surface area and reactivity are crucial. The narrow distribution further suggests effective capping and stabilization by the phytochemicals present in the plant extract used during synthesis [53].

**Figure 4:**
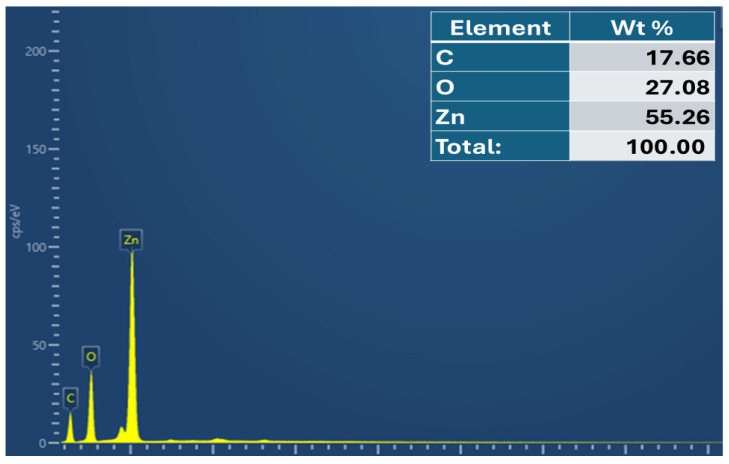
EDS spectrum of Av-ZnO NPs, with an elemental composition table (inset).

**Figure 5:**
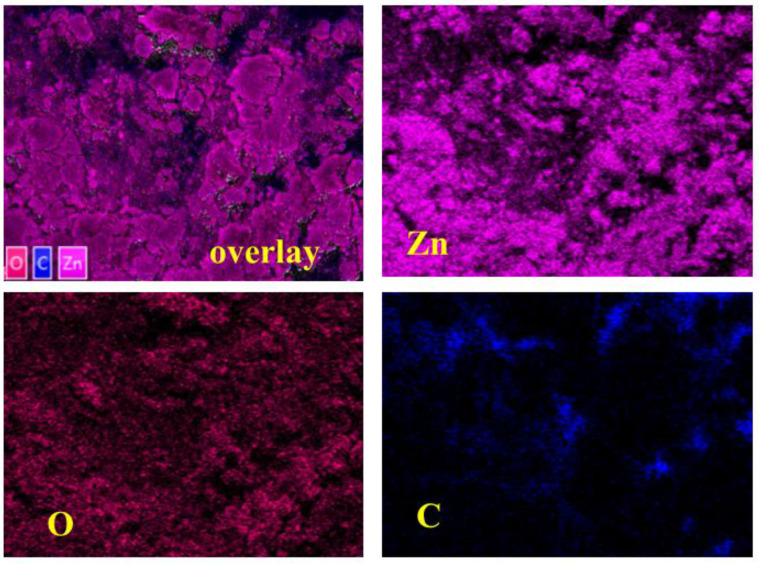
Layered image and color mapping images of elements.

Figure 4 depicts the Av-ZnO NPs’ elemental composition, as well as each element’s weight percentage. The elemental composition was validated by the EDS spectra, which showed a noticeable peak for zinc at 1 keV, demonstrating that zinc was the primary element found in the NP sample. The peak at near 0.5 keV aligns with oxygen. The detection of carbon in EDS is likely due to residual organic matter originating from the plant extract used during synthesis or possibly from the carbon tape employed during SEM sample preparation. This assumption is further supported by the FTIR analysis, which reveals the presence of functional groups such as aromatic phenols and other organic moieties linked to the extract. These observations suggest that plant-derived compounds contributed to both the formation and stabilization of the nanoparticles. The elemental color mapping for each element is demonstrated in Figure 5.

### 2.7. Antimicrobial Activity

**Figure 6:**
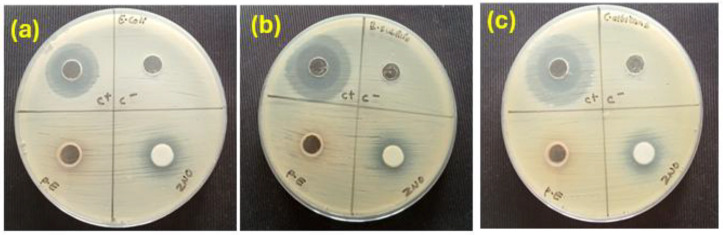
Zones of inhibition displayed by Av extracts with Av-ZnO NPs: (**a**) *E. coli*; (**b**) *B. subtilis*; and (**c**) *C. albicans*.

The ZnO NPs formed with the extract were tested for their antibacterial activity against common ATCC bacterial strains and a fungal strain using the agar well diffusion method (Figure 6). The data are illustrated in Table 4.

Specifically, inhibition zones of 1.3 cm were recorded for both *E. coli* and *B. subtilis*, while *C. albicans* showed a slightly larger zone of inhibition at 1.4 cm, which might be because fungal cells are larger and more structurally complex, providing a larger surface area for interactions with NPs [54]. In contrast, the *Aloe vera* extract alone demonstrated no antimicrobial activity, as no inhibition zones were observed. The lack of efficacy could be due to the use of distilled water as a solvent, which may not have adequately extracted the bioactive chemicals. DMSO was the negative control, while kanamycin (5 mg/mL) served as a positive control. As expected, DMSO exhibited no antimicrobial effect. ZnO NPs kill bacteria through multiple mechanisms. They take in photons from UV or near-UV light, which stimulate electrons and create ROS, such as hydroxyl radicals (^•^OH), superoxide anions (O_2_^•−^), and hydrogen peroxide (H_2_O_2_). Various ROS cause cell death by destroying lipids, proteins, and DNA. ZnO can also produce ROS under visible light due to surface defects [55].

Additionally, ZnO NPs have a positive surface charge that disrupts bacterial membranes, causing the leakage of cellular contents. Their sharp edges physically puncture the cell wall, leading to lysis [56]. Furthermore, by disrupting bacterial enzymes, the zinc ions (Zn^2+^) released by ZnO NPs have an impact on respiration and metabolism. Bacterial antioxidant defenses are overpowered by excessive ROS generation, which results in oxidative damage and programmed cell death. These combined actions make ZnO NPs highly effective against microbes [56].

The green-synthesized ZnO nanoparticles using *Aloe vera* extract exhibited inhibition zones of 13 mm against *E. coli* and 14 mm against *Candida albicans*, compared to 18 mm (kanamycin) and 19 mm (itraconazole) for the positive controls. These results are comparable to those of a previous work [28], which reported zones of 18 mm and 17 mm for ZnO NPs synthesized from *Swertia chirayita*, with the corresponding positive controls showing 26 mm and 25 mm, respectively. When considered relative to their respective positive controls, the antimicrobial performance of the Av-ZnO NPs is comparable, supporting their potential as effective, green-synthesized antibacterial and antifungal agents. Similarly, Haque et al. (2020) reported inhibition zones of 14.5 mm against *E. coli* using ZnO NPs synthesized from *Azadirachta Indica* extract [57]. These comparisons further support the effectiveness of our biosynthesized ZnO nanoparticles.

### 2.8. Assessment of Antioxidant Characteristics

**Figure 7:**
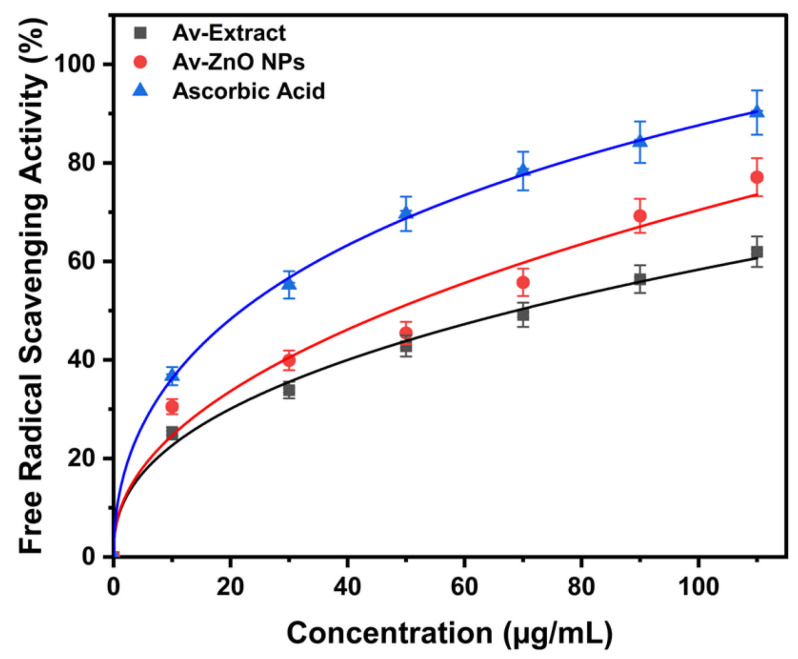
The proportion of free radical scavenging activity evaluated at various concentrations. Data are reported as average ± SD from triplicate experiments (n = 3).

The DPPH assay was employed to assess the free radical scavenging capacity of the *Av* extract and the synthesized NPs. Figure 7 demonstrates that the DPPH radical scavenging activity of the tested compounds increased progressively with rising concentrations. Among them, the Av-ZnO NPs consistently showed higher radical inhibition than the *Av* extract at each concentration, while ascorbic acid displayed the highest activity as a positive control. This trend reflects an enhanced antioxidant capacity due to NP formation and aligns with prior reports [58]. To further quantify the activity, IC_50_ values were determined through nonlinear regression analysis. The IC_50_ values obtained were 62.66 µg/mL for the extract, 38.33 µg/mL for Av-ZnO NPs, and 19.96 µg/mL for ascorbic acid. The low IC_50_ value of the Av-ZnO NPs compared to the extract confirms their enhanced antioxidant efficacy. This is most likely owing to the combined impacts of the material’s large surface area and the bioactive chemicals in the plant extract [59,60].

### 2.9. Photocatalytic MB Dye Degradation

**Figure 8:**
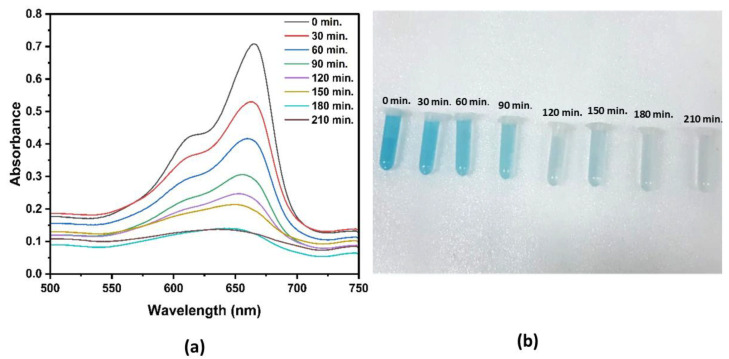
(**a**) Time-resolved UV–vis signal reveals the breakdown process of MB. (**b**) Gradually, MB loses its color over time when exposed to UV light.

The MB degradation process in the synthesized NPs was monitored using UV–vis spectrophotometry, with the absorbance recorded at regular time intervals for a total duration of 210 min, as shown in Figure 8. After 210 min of exposure, there was a notable reduction in the concentration of MB, resulting in degradation efficiency of approximately 82.43%. These results underscore the effective photocatalytic properties of the synthesized NPs. The degradation efficiency observed here aligns with findings from previous studies. For example, a degradation rate of 65% within 180 min was shown in a previous study [61], while other research has reported efficiencies of between 70% and 85% under similar UV light conditions [62]. Overall, these outcomes suggest that green-synthesized ZnO NPs demonstrate competitive, if not superior, photocatalytic effectiveness when under UV irradiation.

**Figure 9:**
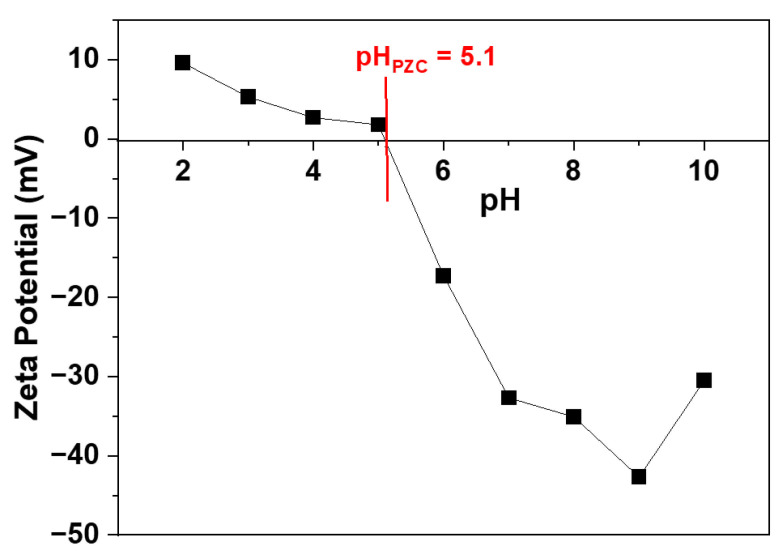
Zeta potential measured at different pH values.

To further understand the stability of the colloidal dispersion, zeta potential measurements were carried out. Colloidal particles exhibiting a high zeta potential (both positive and negative) typically remain stable, i.e., they do not aggregate and flocculate. In this study, the surface charge characteristics of the Av-ZnO nanoparticles were examined using zeta potential analysis over a pH range of 2 to 10 to assess their potential in degrading MB dye. As illustrated in Figure 9, a decrease in zeta potential was observed as the pH increased, and the isoelectric point (pH_PZC_) was found to be 5.1. Since the photocatalytic experiments were performed at a neutral pH (pH 7.2) above the pH_PZC_, the nanoparticle surfaces were negatively charged during the reaction. Given that MB is a positively charged dye, this negative surface potential favored electrostatic interactions, thereby improving the dye’s adsorption onto the catalyst. This enhanced adsorption contributed to the more efficient and accelerated photodegradation of MB under light exposure [63]. Additionally, the presence of a high negative zeta potential at the working pH indicates favorable colloidal stability, minimizing particle aggregation and thus preserving the large surface area and accessibility of active sites [64]. These aspects collectively played a significant role in the enhanced photocatalytic activity observed during the experiments.

**Figure 10:**
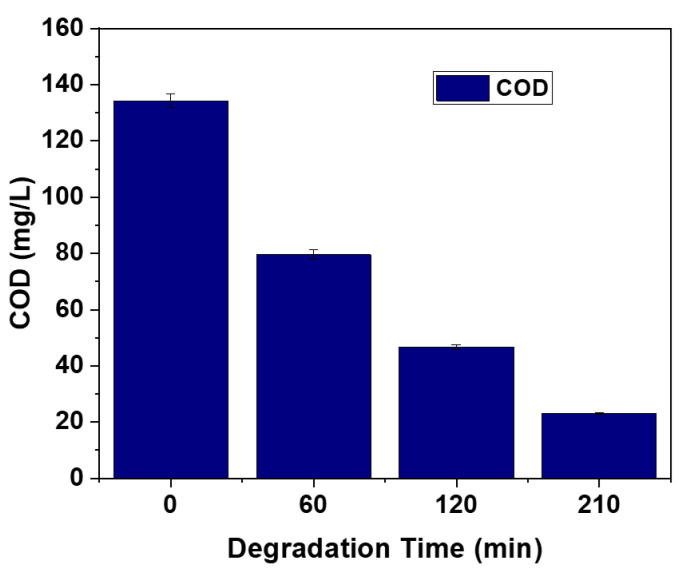
COD study of degraded MB solution.

The chemical oxygen demand (COD) is a reliable indicator in determining the concentrations of oxidizable contaminants in water samples [65]. In this study, the COD values of the MB dye solution were evaluated before and after photocatalytic treatment using the Av-ZnO NPs, and the values were found to be 134.3 mg/L to 23.1 mg/L, respectively (Figure 10). The significant reduction in COD after irradiation confirms the degradation of MB dye, indicating the efficiency of the Av-ZnO NPs in removing organic pollutants. These results align with the findings of Shinde et al. [66], where plant-mediated ZnO nanoparticles effectively degraded dye pollutants under solar light. ZnO NPs are known to generate reactive species that break down complex organic dyes into simpler, non-toxic end products like carbon dioxide and water via mineralization [67]. The degradation process observed here is a typical advanced oxidation process (AOP), where hydroxyl radicals, formed during photocatalysis, are primarily responsible for breaking down organic molecules. Importantly, this occurs without the formation of harmful secondary byproducts such as hydrogen peroxide. The final products are environmentally safe, consisting mainly of CO_2_, H_2_O, and benign mineral residues [68].

**Figure 11:**
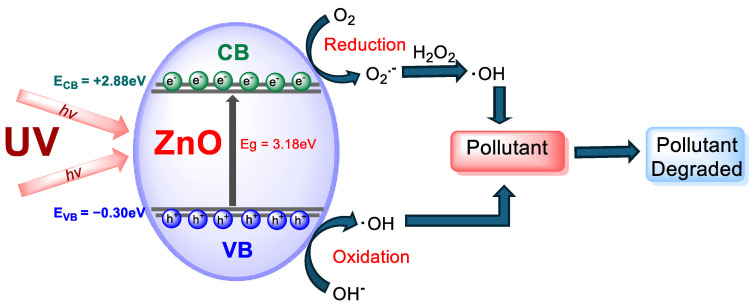
Representation of the photocatalytic mechanism of ZnO NPs.

The main mechanism by which MB breaks down through ZnO NPs is a photocatalytic reaction that generates ROS. When UV light causes ZnO to absorb photons with a band gap energy of ~3.18 eV, it excites the material, generating electron–hole pairs, negatively charged electrons, and positively charged holes. These species help in the formation of the ROS. The holes participate in the oxidation of H_2_O, while the photoexcited electrons reduce O_2_, resulting in the production of hydrogen peroxide (H_2_O_2_), which can occur through either a single-electron transfer stepwise or a direct two-electron transfer, which is part of the oxygen reduction reaction (ORR). The selectivity and efficiency of H_2_O_2_ generation largely depend on how effectively the two-electron pathway is promoted during the ORR. This method of producing H_2_O_2_ is central to the photocatalytic action of NPs, which leads to the generation of the ROS [35]. For organic contaminants like MB to be oxidized and ultimately disintegrated, these reactive species are necessary. Figure 11 shows the proposed mechanism; based on previous studies, it involves the following steps [28,41,69]:

ZnO + hν → ZnO (h^+^ _VB_)/ZnO (e^−^ _CB_)

where h^+^_VB_ and e^−^_CB_ are photoinduced holes and photoelectrons, respectively.

h^+^ + H_2_O → OH^•^ + H^+^

e^−^ + O_2_ → O_2_^•−^

O_2_^•−^ + H^+^ → HO_2_^•^

HO_2_^•^ + HO_2_^•^→ H_2_O_2_ + O_2_

e^−^ + H_2_O_2_ → OH^•^ + OH^−^

O_2_^•−^ + H_2_O_2_ → OH^•^ + OH^−^ + O_2_

OH^•^ + MB → oxidation product → final species.

**Figure 12:**
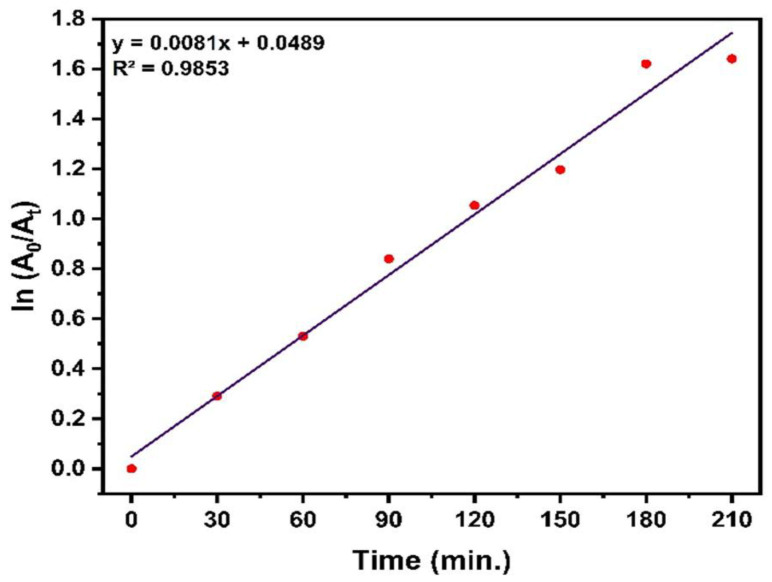
Kinetic plot illustrating the pseudo-first-order breakdown behavior of MB.

Kinetic degradation was assessed, as shown in Figure 12. The linear trend observed in the graph reflects behavior that is consistent with the behavior expected for a pseudo-first-order (PFO) kinetic process, as described by Equation (8):

ln(A_0_/A_t_) = k_t_ (8)

Here, A_0_ represents the initial absorbance, A_t_ is the absorbance at time t, and k denotes the rate constant. The slope of the plot of ln(a_0_/a_t_) vs. time corresponds to the rate constant k, highlighting their direct relationship. The high correlation coefficient (R^2^ = 0.9853) demonstrates the suitability of the PFO model in portraying MB degradation under the applied conditions. The determined rate constant (k) was 0.0081 min^−1^.

The observed degradation efficiency under UV light confirms the photocatalytic potential of the synthesized nanoparticles. However, this study primarily aimed to assess the photocatalytic efficiency under light exposure. Future studies will incorporate additional control experiments, such as reactions in the absence of a catalyst and under dark conditions, to further differentiate photocatalytic effects from photolysis and adsorption.

## 3. Materials and Methods

### 3.1. Plant Components and Chemicals

Deionized water and high-grade reagents were used throughout the laboratory procedures. Sodium hydroxide (NaOH), methanol (CH_3_OH), dimethyl sulfoxide (DMSO), and a zinc nitrate (Zn(NO_3_)_2_·6H_2_O) precursor were sourced from Thermo Fisher Scientific India Pvt., Ltd. (Mumbai, India) L-ascorbic acid was acquired from E. Merck (Bengaluru, India), while 2,2-diphenyl-1-picrylhydrazyl (DPPH) was procured from Sigma Aldrich (Bengaluru, India).

### 3.2. Preparation of Aloe vera Leaf Extracts

To remove contaminants, the *Aloe vera* stem was carefully cleaned with deionized water. After cleaning, the leaves were finely chopped with a knife and blended into a smooth paste using an electric grinder. The *Aloe vera* paste and water, combined in a 1:1 (*w*/*v*) ratio, were maintained at 70 °C for 1 h under continuous magnetic stirring. Throughout this heating process, the solution transitioned from green to a subtle yellow color. Filtration was performed to eliminate undissolved particles, and the purified filtrate was subsequently used in further analyses.

### 3.3. Phytochemical Screening of Extract of Aloe vera

Extracts can be acquired using various methods, with cold percolation and hot Soxhlet extraction being among the most utilized. One gram of plant extract was solubilized in 100 milliliters of methanol to produce a stock solution. The identification of secondary metabolites was conducted using standard chemical assays, including the alkaline reagent test, Wagner’s test, the frothing/foam test, the Salkowski test, the ferric chloride reagent test, and the Benedict test, with minor adjustments based on guidelines found in the literature [30].

### 3.4. Av-ZnO Synthesis

**Figure 13:**
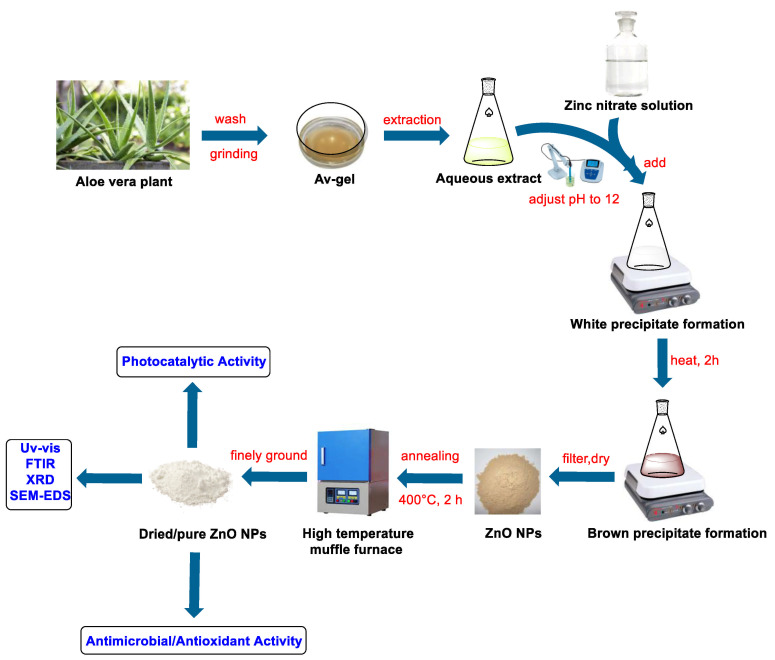
A representation of the ZnO synthesis process.

The previously described process was slightly modified to synthesize the Av-ZnO NPs [70]. Initially, 50 mL of plant extract was prepared in a beaker. The pH was maintained at 12 by the dropwise addition of 0.1 M NaOH. The solution was stirred continuously with the help of a stirrer. Then, a 0.1 M solution of Zn(NO_3_)_2_·6H_2_O was added slowly when the temperature reached 60 °C. This addition led to the formation of a white solid substance. The reaction mixture was heated for a further two hours at 70 °C, which converted the white precipitate to a brown color. After cooling, the mixture was strained, and the solid residue was transferred to a beaker. The solid residue was heated for one hour to ensure the complete elimination of impurities. Following a second filtration step, the solid was isolated and dried under sunlight. To ensure complete drying and eliminate any volatile substances, the sample was further heated at 80 °C in an oven. Lastly, the ZnO was annealed at 400 °C for two hours, resulting in white Av-ZnO NPs. The resulting material was roughly crushed using a grinder machine and then sealed in an airproof vessel for future examination. Figure 13 shows a diagram of the Av-ZnO NP synthesis.

### 3.5. Characterization of Av-ZnO NPs

A UV–vis spectrophotometer (Cary 60, Agilent Technologies, Santa Clara, CA, USA) was used to study how the ZnO NPs absorbed light. Before testing, the particles were mixed with ethanol and treated with ultrasound for 30 min to help them spread evenly. The absorbance was then measured across a spectrum from 200 to 800 nanometers. Ethanol served as the blank reference throughout the experiment. To investigate the functional groups present in the plant extracts, FTIR on the Av-ZnO NPs was performed using PerkinElmer Spectrum IR (Version 10.6.2), scanning between 4000 and 400 cm^−1^. The XRD analysis of the Av-ZnO NPs was performed using a Bruker D2 Phaser diffractometer (Karlsruhe, Germany) with monochromatic Cu Kα radiation (λ = 1.5406 Å). The scanning system was operated in a 2θ range of 20° to 80°. Scherrer’s Equation (9) was applied to measure the size of the Av-ZnO NPs in powder form [29,71]:

(9) $$D=\frac{k\cdot \lambda }{\beta cos\theta }$$

An FE-SEM instrument (Hitachi, Japan) equipped with an EDS instrument was used to analyze the surface morphology and elemental composition of the NPs. The zeta potential of the ZnO NPs at different pH values was analyzed to determine the change in surface charge using a nanoparticle analyzer (Horiba Scientific SZ-100V2, Kyoto, Japan).

### 3.6. Preparation of Microbial Culture Media

First, 13 g of LB solid powder (Sisco Research Laboratories Pvt., Ltd., Mumbai, India) was solubilized in one liter of deionized water to create the LB medium. The mixture was then autoclaved at 121 °C and 15 psi for 25 min to ensure sterilization. Following autoclaving, the medium was cooled to around 40 °C before being used in subsequent procedures. Following aseptic distribution, 5 mL was added to each of several 15 mL falcon tubes that had been sterilized. Bacterial co-culturing was performed using the resultant medium, with separate inoculations in each tube and a 24 h incubation period.

#### 3.6.1. Preparation of MH Media Plates

Mueller–Hinton agar (MHA) plates were created by mixing 39 g of MH agar powder, sourced from Sisco Research Laboratories Pvt., Ltd., India, with one liter of deionized water. The mixture was subjected to autoclaving at 121 °C and 15 psi for 25 min to guarantee complete sterilization. Once sterilized, the medium was cooled to about 40 °C before being distributed into sterile Petri dishes, with each dish receiving 25 mL of the medium.

#### 3.6.2. Antimicrobial Assay Protocol

The prepared culture media remained refrigerated until testing was performed. For the antimicrobial assay, the agar plates were labeled with the names of the samples. Then, 100 µL of fresh bacterial culture was evenly applied to the agar using a sterile swab. Sterilized pipette tips were used to create 9-mm-diameter wells with a 3 mm depth in the agar, allowing for the placement of both test samples and standards. To reach a concentration of 100 mg/mL, 100 µL of test sample was mixed with DMSO in each well. A standard kanamycin solution for bacterial strains and itraconazole for fungal strains were used as positive controls. The plates were incubated at 37 °C for 24 h. The diameter of the zone surrounding each well, devoid of bacterial growth or antimicrobial activity, was assessed following incubation.

### 3.7. Evaluation of Antioxidant Potential Through DPPH Radical Scavenging Assay

The antioxidant activity of the Av-ZnO NPs and Av extract was evaluated using the 2,2-diphenyl-1-picrylhydrazyl (DPPH) free radical scavenging method, following a predesigned protocol, with slight modifications [72,73]. A stock solution of the extract and Av-ZnO NPs was solubilized in 50% DMSO. Likewise, the dilution of standard ascorbic acid was carried out from 1 mg/mL stock solution. The NPs were vortexed for 5 min before the serial dilution of the stock solution and were diluted to the final concentrations of 10, 30, 50, 70, 90, and 110 µg/mL. A 96-well microplate was filled with 100 µL of each test solution in triplicate, through the use of 100 µL of a DPPH solution containing 0.1 mM. Blanks containing the solvent without DPPH were also included. In the dark, the microplates were incubated for half an hour following the initial reading, and the solution’s signal at 517 nm was recorded. Using the measured absorbance values, the extract and Av-ZnO NPs’ percentages of scavenging activity at various concentrations were determined. A positive control in the form of ascorbic acid was used. Data analysis was carried out using Microsoft Excel and Origin Pro 2025 (Learning Edition) to calculate IC_50_ values and generate dose–response curves.

### 3.8. Photocatalytic Study

The degradation was assessed using the photocatalysis of the NPs. MB dye was placed in a UV cabinet (Agilent Technologies, Cary 60) and exposed to UV light (wavelength 365 nm). A stock solution of MB (100 mg/L) was prepared by dissolving 0.1 g of MB dye in 1 L of distilled water. To this MB stock solution, 2 g/L Av-ZnO NPs was added. An adsorption–desorption reaction was established by shaking the mixture in the dark for 30 min. The reaction mixture was exposed to UV light and continuously agitated with a magnetic stirrer. Every 30 min, 4 mL of the mixture was withdrawn and centrifuged at 5000 rpm for 3 min to separate the catalyst particles. The resulting supernatant was then analyzed using a UV–vis spectrophotometer (Cary 60, Agilent Technology, Santa Clara, CA, USA) within 500 to 750 nm. The absorbance at 664 nm, corresponding to MB, was recorded to assess the degradation efficiency. The % deterioration was calculated using Equation (10) [39]:

(10) $$\;\mathrm{Percentage}\;\mathrm{of}\;\mathrm{degradation}=\frac{A_{0}-At\;}{A_{0}}\times 100$$

Here, *A*_0_ indicates the initial absorbance of the MB solution, while *A*_t_ specifies the absorbance obtained at a certain time *t*.

The mineralization efficiency of the Av-ZnO NPs was evaluated by analyzing the COD of the MB dye solution before and after exposure to the photocatalytic treatment. The COD measurements were conducted using a standard method, namely 5220 B-COD: closed reflux, titrimetric method, from the APHA-AWWA-WEF, 24th edition [74].

## 4. Conclusions

In this work, ZnO NPs were successfully synthesized via a sustainable biosynthesis approach using *Aloe vera* extract. An absorption spectrum at 350 nm from UV–vis spectroscopy confirmed the existence of ZnO NPs. Similarly, the Tauc plot technique yielded a band gap energy value of 3.18 eV. From the FTIR analysis, the functional groups in the NPs were identified, whereas XRD examination verified the wurtzite structure and crystalline nature of the NPs. FE-SEM displayed a spherical shape with the minor agglomeration of the particles. The evaluation of the antimicrobial activity indicated that the Av-ZnO NPs had moderate inhibitory effects, whereas *Aloe vera* extract did not demonstrate antimicrobial properties. The DPPH assay confirmed that the Av-ZnO NPs possessed superior antioxidant activity compared to the extract, highlighting their potential as effective free radical scavengers. The degradation of MB dye was monitored as part of the photocatalytic performance analysis using exposure to UV light, achieving degradation effectiveness of 82.43% in 210 min, which was linked to the production of ROS from electron–hole pair excitation. These ZnO NPs exhibit strong promise for antimicrobial uses and for the breakdown of organic pollutants through photocatalysis. The high photocatalytic activity of Av-ZnO under UV light suggests its potential for wastewater treatment, particularly in the degradation of organic dyes. The significant antimicrobial activity indicates its suitability for use in antibacterial coatings, wound dressings, and packaging materials. Additionally, the antioxidant capacity of the ZnO nanoparticles points to potential applications in skincare formulations aimed at combating oxidative stress. While *Aloe vera* provides excellent reduction and stabilization properties, the batch-to-batch variability in the extract composition may affect its reproducibility. This study focused on model dyes and laboratory-scale testing; real-world applications will require further pilot studies in environmental or biomedical settings. Future research could aim to scale the synthesis process and refine it for industrial use.

## Figures and Tables

**Table 1:** Phytochemical screening of *Aloe vera* extract.

S.N.	Secondary Metabolites	Test	Result
1.	Flavonoids	Alkaline reagent test	+
2.	Alkaloids	Wagner test	+
3.	Saponins	Froth test	+
4.	Glycosides	Keller–Kiliani test	−
5.	Tannins	Ferric chloride test	+
6.	Quinones	Conc. HCl test	−
7.	Anthraquinones	Borntrager’s test	+
8.	Phenols	Ferric chloride test	+

+ indicates presence and − indicates absence of phytochemicals.

**Table 2:** The structural parameters of the ZnO NPs, including δ, ε, and SF.

θ	FWHM	Miller Index	Crystallite Size (D), nm	d-Spacing [Å]	δ (nm)	ε	SF	Rel. Int. [%]
15.86	0.78838	(1 0 0)	10.48	2.81718	0.00911	0.00331	0.02065	65.05
17.14	0.78838	(0 0 2)	10.54	2.60650	0.00899	0.00328	0.01980	51.46
18.09	0.78838	(1 0 1)	10.60	2.47842	0.00890	0.00327	0.01926	100
23.70	1.18257	(1 0 2)	7.34	1.91322	0.01856	0.00473	0.02491	14.62
28.26	0.78838	(1 1 0)	11.44	1.62650	0.00764	0.00303	0.01492	41.47
31.40	0.98548	(1 0 3)	9.44	1.47896	0.01122	0.00367	0.01759	22.46
33.14	0.59129	(2 0 0)	16.05	1.40859	0.00388	0.00216	0.01021	4.34
33.92	0.59129	(1 1 2)	16.19	1.40859	0.00381	0.00214	0.01006	27.16
34.58	0.59129	(2 0 1)	16.32	1.35982	0.00375	0.00212	0.00996	10.58
36.18	0.78838	(0 0 4)	12.48	1.30325	0.00642	0.00277	0.01293	1.79
38.38	0.78838	(2 0 2)	12.85	1.23921	0.00606	0.00269	0.01234	1.68

**Table 3:** Structural parameter comparison of ZnO NPs synthesized via different methods.

Method	Crystallite Size (nm)	Dislocation Density (δ, nm^−2^)	Strain (ε)	Reference
Sol–gel	50.76–69.65	0.21–0.39	0.89–1.21	[48]
Hydrothermal	31–51	0.00384–0.0056	0.015–1.59	[49]
Precipitation	23.5 ± 6.9	0.0018	0.347	[50]
Microwave	20–22	0.0021–0.00285	0.0388–0.0438	[51]
Green synthesis	6–18	0.003–0.027	0.00202–0.00598	[46]
Coprecipitation	16.81–31.80	0.001–0.0035	0.0049–0.008	[52]
Green synthesis	7.34–16.32	0.00375–0.01856	0.00212–0.00473	This work

**Table 4:** Zones of inhibition of Av-ZnO NPs, plant extract, and positive control were measured against various microbial strains.

Microbes	Reference Culture	Type	Positive Control (C^+^), cm	Plant Extract, cm	Av-ZnO NPs, cm
*Escherichia coli*	ATCC8739	Gram −ve	1.8	0	1.3
*Bacillus subtilis*	ATCC 6051	Gram +ve	2.0	0	1.3
*Candida albicans*	ATCC2091	Fungi	1.9	0	1.4

## Data Availability

Data are contained within the article.
